# Fermented Noni Polysaccharides and Immune-Related Biomarkers in Adults with Recurrent URTIs: A Randomized, Double-Blind, Placebo-Controlled Trial

**DOI:** 10.3390/nu18111691

**Published:** 2026-05-26

**Authors:** Seon-Mi Shin, Seong-Hwan Park, Seon-Gyu Bae, Eun-Young Park, Jae-Yeon Lee, Hee-Yeon Kwon, Im-Joung La, Sang-Jun Youn, Yong Choi, Yeong-Eun Choi, Do-Hee Kim, Sun-Young Park, Cheol Moon, Tae-Yeon Kim

**Affiliations:** 1 Department of Internal Medicine, College of Korean Medicine, Semyung University, 65 Semyung-ro, Jecheon-si 27136, Republic of Korea; dollkong43@naver.com (S.-H.P.); qotjsrb6691@daum.net (S.-G.B.); 2 R&D Center, NSTbio Co., Ltd., 32 Songdogwahak-ro, Yeonsu-gu, Incheon 21984, Republic of Korea; pey@nstbio.co.kr (E.-Y.P.); jyleebio@nstbio.co.kr (J.-Y.L.); khy@nstbio.co.kr (H.-Y.K.); 3 Atomy R&D Center, Atomy Co., Ltd., 3526 Charyeong-ro, Jeongan-myeon, Gongju-si 32511, Republic of Korea; imjna@atomyorot.kr; 4 RnBS Corporation, 11 Nonhyeon-ro 155-gil, Gangnam-gu, Seoul 06032, Republic of Korea; sjyoun@rnbs.co.kr (S.-J.Y.); choiy@rnbs.co.kr (Y.C.); 5 Redox BioMedicine Laboratory, Wonju College of Medicine, Yonsei University, 1 Yonseidae-gil, Wonju 26493, Republic of Korea; yeongeun4833@gmail.com; 6 Department of Physiology, College of Korean Medicine, Semyung University, 65 Semyung-ro, Jecheon-si 27136, Republic of Korea; monkey1507@naver.com (D.-H.K.); blbee@semyung.ac.kr (S.-Y.P.); 7 Department of Clinical Laboratory Science, Semyung University, 65 Semyung-ro, Jecheon-si 27136, Republic of Korea; moonc72@naver.com; 8 Department of Pathology, College of Korean Medicine, Semyung University, 65 Semyung-ro, Jecheon-si 27136, Republic of Korea; violet805@semyung.ac.kr

**Keywords:** fermented noni, polysaccharides, natural killer cell activity, immune system, randomized controlled trial

## Abstract

Background: Fermented polysaccharides derived from *Morinda citrifolia* (noni) have been suggested to modulate innate immune responses, but clinical evidence remains limited. Objectives: This randomized, double-blind, placebo-controlled trial evaluated the effects of fermented noni polysaccharides on natural killer (NK) cell activity and immune-related biomarkers in adults with recurrent upper respiratory tract infections (URTIs). Methods: A total of 100 adults aged 40 to <75 years with a documented history of ≥2 episodes of upper respiratory tract infection in the prior 12 months were randomly assigned to receive fermented noni polysaccharides (487.5 mg/tablet, two tablets once daily; 975 mg/day of FNP extract) or a matched placebo for 8 weeks. The primary endpoint was the change in NK cell activity at effector-to-target (E:T) ratios of 50:1, 25:1, and 12.5:1, assessed using K562 NK-sensitive target cells. Secondary endpoints included circulating cytokines (IFN-γ, TNF-α, IL-2, IL-6, IL-10, IL-12, IL-1β) and immunoglobulin G (IgG). Eighty-four participants (43 treatment, 41 placebo) were included in the modified intention-to-treat/full analysis set (mITT/FAS); 81 participants (41/40) constituted the per-protocol set (PPS). Primary efficacy was analyzed in the mITT/FAS. This trial was retrospectively registered at CRiS (KCT0011316) after trial completion; the IRB-approved protocol was finalised before enrolment and remained unchanged thereafter. Results: NK cell activity in the treatment group increased from baseline at all three E:T ratios, whereas it slightly decreased in the placebo group. Adjusted between-group LS mean differences (95% CI) were +8.94 (−0.61, 18.50; *p* = 0.066) at E:T 50:1, +7.68 (−1.14, 16.50; *p* = 0.087) at 25:1, and +3.29 (−2.95, 9.54; *p* = 0.145) at 12.5:1, all favouring treatment but not reaching the conventional threshold for significance in the mITT/FAS. Prespecified PPS sensitivity analyses reached significance at E:T 50:1 (+11.03; *p* = 0.025) and 25:1 (+9.94; *p* = 0.028). Selected cytokines (IFN-γ, IL-2, IL-6, IL-10, IL-1β) increased to a greater extent in the treatment group than in the placebo group, whereas TNF-α, IL-12, and IgG were unchanged. URTI incidence at week 4, week 8, and cumulatively did not differ significantly between groups. The intervention was well tolerated, with no serious adverse events and no treatment-related discontinuations. Conclusions: Compared with placebo, fermented noni polysaccharide supplementation showed numerically greater increases in NK cell activity at all three E:T ratios (50:1, 25:1, and 12.5:1) in the primary mITT/FAS analysis, although these between-group differences did not reach statistical significance. Prespecified per-protocol set (PPS) sensitivity analyses showed significant between-group differences at E:T ratios of 50:1 and 25:1. The treatment group also showed greater increases in selected cytokines (IFN-γ, IL-2, IL-6, IL-10) relative to placebo. No significant between-group differences were observed in URTI incidence, IgG, GARS, WBC, or leukocyte subset proportions. These exploratory biomarker findings, in the absence of safety signals, suggest a possible immunomodulatory profile but do not establish clinical efficacy. Confirmation in larger, prospectively registered trials with clinically adjudicated infection-related endpoints is warranted.

## 1. Introduction

Impaired immune function increases susceptibility to infections and chronic inflammatory conditions and may delay recovery and accelerate aging-related processes [1]. Contemporary risk factors for impaired immune function that disrupt immune homeostasis include aging, persistent psychological stress, and insufficient sleep. Moreover, environmental exposures, including particulate matter, further aggravate the respiratory and allergic disease burden [1,2]. Age-related immunosenescence, characterized by declining NK cell function and altered cytokine profiles, further compounds infection susceptibility in older populations [3]. There is growing interest in preventive, safe, and evidence-based nutritional strategies that support immune resilience because broad pharmacologic approaches to “enhance immunity” are limited and may be constrained by adverse effects [4]. Fermented foods are increasingly studied as dietary strategies that can modulate the gut microbiome and immune function [5]. Microbial fermentation can generate novel bioactive metabolites and modify the structural characteristics of polysaccharides, potentially enhancing their immunological properties [6]. Among botanical sources of immunomodulatory polysaccharides, noni (*Morinda citrifolia*) is of particular interest as a candidate functional-food ingredient, and is considered in the next paragraph.

Noni (*Morinda citrifolia*) is a traditional botanical resource found in Polynesia, India, and parts of Asia that contains diverse bioactive constituents, including phenolics, alkaloids, lignans, fatty acid esters, organic acids, vitamins, and minerals [7,8]. Among these, iridoid glycosides and polysaccharides have attracted particular attention for their immunomodulatory potential [9]. Noni is further characterized by remarkable ecological adaptability and high cultivation potential, allowing year-round harvesting in various tropical and subtropical regions [10,11,12]. Recent reviews have highlighted that noni contains over 200 bioactive phytochemicals—including iridoids, flavonoids, and polysaccharides—that exhibit antioxidant, anti-inflammatory, and immunomodulatory activities [10,11,12]. Owing to these properties, industrial application of noni has expanded beyond traditional use into pharmaceutical, nutraceutical, and cosmeceutical sectors, indicating significant global market potential [10,11,12].

Plant-derived polysaccharides are of particular interest as immunomodulators because their structural features can influence innate immune signalling through interactions with pattern-recognition receptors on immune cells, thereby shaping cytokine responses and effector-cell function [13,14]. Fermentation may further modify polysaccharide composition and bioactivity [5]. Preclinical studies of fermented noni polysaccharides have suggested immune-activating effects, including increased nitric oxide production, upregulation of inflammatory mediators such as interleukin (IL)-6, IL-1β, and tumour necrosis factor (TNF)-α, increased cyclooxygenase-2 and inducible nitric oxide synthase expression, and enhanced natural killer (NK) cell cytotoxic activity [15]. In addition, increases in Th1- and Th2-associated cytokines and NK activation-related immune cell subsets have been reported, supporting the biological plausibility of immune-support effects [15].

NK cells are cytotoxic lymphocytes central to early innate immune defence against virus-infected and transformed cells, and functional NK cell activity assays provide a clinically relevant readout of innate cytotoxic capacity [16]. Innate immune cells such as macrophages also coordinate immune responses by producing cytokines that link innate recognition to adaptive immune activation [13]. On the basis of the preclinical evidence summarised above and the central role of NK cytotoxic activity as a clinically relevant biomarker of innate immune competence in middle-aged and older adults, we conducted this randomised, double-blind, placebo-controlled trial to evaluate whether 8-week supplementation with fermented noni polysaccharide extract modifies NK cell cytotoxic activity and circulating immune-related cytokines in adults with a documented history of recurrent upper respiratory tract infections (URTIs). Secondary aims were to characterise the directional pattern of cytokine responses, to assess safety and tolerability, and to explore changes in clinically relevant descriptive parameters including URTI occurrence and perceived stress.

## 2. Materials and Methods

### 2.1. Study Design and Ethics

This randomized, double-blind, placebo-controlled, parallel-group clinical trial was conducted at Semyung University Jecheon Korean Medicine Hospital (Jecheon, Republic of Korea). The protocol was approved by the Institutional Review Board of Semyung University Jecheon Korean Medicine Hospital (SMJOH-2023-13; 29 December 2023). Written informed consent was obtained from all participants before any study procedures. The trial was conducted in accordance with the Declaration of Helsinki, ICH-GCP, applicable national regulations, and reported in accordance with CONSORT guidance. The intervention period was 8 weeks and comprised four scheduled on-site visits: screening (Visit 1), baseline (Visit 2), mid-intervention at week 4 (Visit 3), and end of intervention at week 8 (Visit 4) (Figure S1). At each visit, participants underwent protocol-specified assessments, including review of medical history and concomitant medications, vital signs, compliance checks by tablet count, and collection of efficacy and safety endpoints.

The trial was retrospectively registered at the Clinical Research Information Service (CRiS; https://cris.nih.go.kr) under registration number KCT0011316 on 12 December 2025—that is, after the first participant was enrolled on 1 February 2024 and after the last participant visit on 7 January 2025. The authors acknowledge that this registration timing does not conform to the ICMJE recommendation for prospective registration before enrolment of the first participant. Nevertheless, the study protocol—including eligibility criteria, sample size, randomisation procedure, blinding, intervention regimen, primary and secondary outcomes, and statistical analysis plan—had been finalised and prospectively approved by the IRB before recruitment began, and no protocol amendments were made between IRB approval and the end of the trial. The delay in trial registration was due to administrative oversight on the part of the investigators and was not influenced by any post hoc consideration of the study findings. Information that was registered in CRiS is fully consistent with the IRB-approved protocol and with the data presented in this manuscript. This limitation is also acknowledged in the Discussion.

### 2.2. Participants and Eligibility

Participants were recruited via institutional advertisements and screened in the internal medicine department. In this study, “recurrent URTI” was defined operationally as a documented history of ≥2 episodes of upper respiratory tract infection—including the common cold, tonsillitis, pharyngitis, laryngitis, sinusitis, otitis media, or rhinitis—within the 12 months prior to screening. The principal inclusion criteria were: men and women aged 40 to <75 years; peripheral white blood cell count at screening between 4.0 and 8.0 × 10^3^ cells/μL; and provision of written informed consent. Principal exclusion criteria comprised: any condition that may affect immune function (autoimmune disease, malignancy, active hepatitis, systemic inflammation, marked malnutrition); recent use (within 1 month) of immune-supportive supplements; recent use (within 3 months) of immunomodulating medications, vaccination, or COVID-19 infection; uncontrolled hypertension or diabetes; severe organ dysfunction; recent cardiovascular events; gastrointestinal conditions affecting absorption; thyroid dysfunction; heavy alcohol intake or smoking; pregnancy or lactation; and known hypersensitivity to any component of the investigational product. Full inclusion and exclusion criteria are listed in Table S1. Eligibility was determined according to predefined inclusion and exclusion criteria (Table S1). Participants were required to complete a washout period for prohibited products, including immune-related health functional foods or supplements taken within 1 month before screening (e.g., vitamins, probiotics, ginseng, red ginseng, and other immune-enhancing supplements), and medications taken within 3 months before screening that could affect immune function (e.g., immunosuppressants, antibiotics, non-steroidal anti-inflammatory drugs, steroid medications, and antihistamines). Vaccination within 3 months prior to screening was also not permitted. Eligibility was reconfirmed at baseline prior to randomization. In the mITT/FAS, mean age was 50.42 ± 7.96 years in the treatment group and 50.66 ± 7.39 years in the placebo group (*p* = 0.7811, Wilcoxon rank-sum test); women comprised 86.05% (37/43) of the treatment group and 80.49% (33/41) of the placebo group (*p* = 0.4944, χ^2^ test). Detailed baseline demographic and clinical characteristics are presented in Table 1.

### 2.3. Intervention and Compliance

The fermented noni polysaccharide (FNP) was prepared following a standardized procedure as previously described by Choi et al. [15], with minor modifications. Briefly, *Morinda citrifolia* fruits were harvested, washed, and dewatered before being quick-frozen at −40 °C for storage. The frozen fruits were thawed and subsequently inoculated with 0.2% (*w*/*w*) *Levilactobacillus brevis* NST707. Fermentation was carried out at 34 ± 4 °C for 14 days. After fermentation, the material was pressed, filtered through an 80-mesh filter, and concentrated. The polysaccharide fraction was then isolated by ethanol precipitation (1:4, *v*/*v*). After precipitation, the isolate was re-dissolved in purified water, further concentrated, sterilized, and freeze-dried to obtain the final FNP powder.

Galacturonic acid (GalA), the principal acidic monosaccharide of pectic/uronic acid–containing plant polysaccharides, was selected as a representative marker of the FNP fraction. GalA content was determined by HPLC after acid hydrolysis (2 M trifluoroacetic acid, 100 °C, 8 h) and 1-phenyl-3-methyl-5-pyrazolone (PMP) derivatization, using a Shimadzu HPLC system (LC-20AC pumps and SPD-M20A detector; Shimadzu Corporation, Kyoto, Japan) operated with LCsolution software version 1.25 (Shimadzu Corporation, Kyoto, Japan), equipped with a Waters XBridge C18 column (4.6 × 250 mm, 5 μm; Waters Corporation, Milford, MA, USA). Mobile phases were (A) 10% acetonitrile in 100 mM sodium acetate buffer (pH 5.0) and (B) 20% acetonitrile in the same buffer; gradient elution started at A:B 50:50 (*v*/*v*) for 2 min, then immediately shifted to 100% B and held to 50 min, at 1.0 mL/min and 40 °C, with UV detection at 245 nm and a 10 μL injection volume. Under these conditions, the GalA content of the FNP extract was 76.10 mg/g (specification ± 20%) (Figure S2).

Participants were allocated to receive either fermented noni polysaccharide (FNP) extract (Vitalbos; NSTbio Co., Ltd., Incheon, Republic of Korea) or a matched placebo for 8 weeks. The investigational product and placebo were indistinguishable in appearance, taste, and packaging (Figure S2). Participants were instructed to maintain their usual diet and physical activity patterns throughout the trial and to avoid new immune-related supplements or medications. Each tablet contained 487.5 mg of FNP extract, and participants received two tablets once daily before breakfast with water, providing a daily intake of 975 mg of FNP extract (487.5 mg/tablet × 2 tablets/day), consistent with the publicly registered protocol (CRiS KCT0011316). The placebo tablets contained lactose, microcrystalline cellulose, food colouring, silicon dioxide, and magnesium stearate as inactive excipients without any FNP, and were matched to the FNP tablets in size, shape, weight, colour, taste, and packaging to preserve blinding.

Study products were dispensed at baseline (Visit 2) and at the mid-intervention visit (Visit 3, week 4). Returned tablets were counted at weeks 4 and 8 to assess adherence; participants who returned >20% of the dispensed tablets at any visit were flagged for protocol-deviation review. Adherence was reported as the percentage of expected tablets actually consumed, calculated as (dispensed − returned)/expected × 100%.

### 2.4. Randomization and Blinding

Participants were randomized using a stratified block randomization design with age group (40–49, 50–64, >64 years) as the stratification factor. The random sequence was generated by an independent statistician using a SAS software version 9.4 (SAS Institute Inc., Cary, NC, USA), with a fixed block size of 4 and a prespecified seed to ensure reproducibility. Randomization was implemented via a secure web-based Interactive Web Response System (IWRS), which ensured allocation concealment by releasing treatment assignments only after eligibility confirmation and enrollment. Participants, investigators, study staff, IP managers, outcome assessors, monitors, and the statistician were blinded to treatment allocation. The investigational product and placebo were identical in packaging and labeling and were identified only by unique IP numbers. The randomization code and IP list were maintained by an independent unblinded statistician and were not disclosed until database lock and completion of statistical analysis, except in medical emergencies.

### 2.5. Outcomes

The primary outcome was the change from baseline to week 8 in NK cell activity measured at effector-to-target (E:T) ratios of 50:1, 25:1, and 12.5:1. NK cell activity was assessed at baseline (visit 2) and week 8 (visit 4) using a cytotoxicity-based assay. Peripheral venous blood was collected from the forearm vein using standardized phlebotomy procedures designed to minimize hemolysis and clotting. Effector cells were isolated from blood-derived mononuclear cells and co-cultured with NK-sensitive target cells at predefined E:T ratios of 50:1, 25:1, and 12.5:1. The NK-sensitive target cells used in this assay were K562 cells (KCBL No. 10243, Korean Cell Line Bank, Seoul, Republic of Korea), the standard NK-sensitive target cell line in clinical NK cytotoxicity assays [17,18]. NK cell activity was expressed as the percentage of specific lysis at each E:T ratio. Cytotoxic activity was quantified using an LDH cytotoxicity assay kit (Dojindo, Kumamoto, Japan) according to the manufacturer’s instructions. All NK cell activity results generated by the analytical laboratory were transferred to the electronic case report form (eCRF) after assay completion.

Secondary outcomes included changes from baseline to week 8 in circulating cytokines (IFN-γ, TNF-α, IL-2, IL-6, IL-10, IL-12, and IL-1β) and immunoglobulin G (IgG). Plasma samples for cytokine and IgG measurements were stored at −80 °C until analysis. Cytokine concentrations were quantified using the Human ProcartaPlex™ MIX&Match 7-plex panel (ThermoFisher Scientific, Waltham, MA, USA) according to the manufacturer’s instructions, and data acquisition was performed using a Luminex™ 200 system (ThermoFisher Scientific, Waltham, MA, USA). IgG concentrations were determined using an ELISA kit (ThermoFisher Scientific, Waltham, MA, USA; cat. no. BMS2091TEN). Data acquisition and initial analyses were performed using MILLIPLEX Analyst software version 5.1 (Merck Millipore, Burlington, MA, USA) for multiplex cytokine analyses and SoftMax Pro 5.4 (Molecular Devices, Sunnyvale, CA, USA) for the IgG ELISA. Values below the lower limit of quantification (LLOQ) were assigned a value of LLOQ/2 for statistical analyses, whereas samples above the upper limit of quantification (ULOQ) were reanalyzed after appropriate dilution.

Exploratory outcomes included URTI occurrence during the intervention period, total white blood cell (WBC) count and differential percentages (neutrophils and lymphocytes), and perceived stress assessed using the General Assessment of Recent Stress (GARS). WBC count and differential percentages were assessed at baseline and week 8, and GARS was assessed at baseline, week 4, and week 8. Participants were instructed to maintain their usual diet and physical activity throughout the study period. Safety outcomes included adverse events, vital signs, and clinical laboratory tests.

### 2.6. Safety Monitoring

Safety was assessed throughout the trial by monitoring adverse events (AEs), vital signs, and clinical laboratory tests. Hematology, serum chemistry, and urinalysis were performed at baseline and at the end of the 8-week intervention using automated analyzers and standard methods at the study site’s certified laboratory. Laboratory results were reviewed for clinically meaningful abnormalities, and any abnormal findings were evaluated by investigators in the context of medical history and concomitant medications.

### 2.7. Sample Size

The sample size was calculated based on the primary outcome, defined as the between-group difference in the change in NK cell activity from baseline to week 8. Assumptions were derived from a previous clinical study [19]. A two-sided significance level (*α*) of 0.05 and a statistical power (1 − *β*) of 88% were applied. The expected mean change in NK cell activity was assumed to be 8.1% in the intervention group and 3.3% in the placebo group, yielding an anticipated mean difference (Δ) of 4.8 percentage points. The pooled standard deviation (*σ*) was assumed to be 6.8. For a two-sample comparison of means, the required sample size per group was calculated using the following formula:

*n* = (*Z*_1_ − *α*/_2_ + *Z*_1_ − *β*)^2^ × 2*σ*^2^/Δ^2^

Using Z_1_ − *α*/_2_ = 1.96, Z_1_ − *β* = 1.175 (corresponding to 88% power), *σ* = 6.8, and Δ = 4.8, the minimum sample size was approximately 40 participants per group. Assuming a dropout rate of approximately 20%, a total of 100 participants (50 per group) were planned for enrollment.

### 2.8. Statistical Analysis

All statistical analyses were performed using two-sided tests with a significance level of 0.05. Continuous variables are presented as mean ± standard deviation (SD) unless otherwise specified, and categorical variables are presented as number (%).

The primary efficacy analysis was performed in the modified intention-to-treat/full analysis set (mITT/FAS), defined as all randomized participants who consumed at least one dose of the study product and had at least one post-baseline assessment for the relevant endpoint. The per-protocol set (PPS), defined as participants who completed the study without major protocol deviations and with adequate compliance, was analyzed as a prespecified sensitivity analysis. Safety analyses were conducted in the safety analysis set, which included all participants who consumed at least one dose of study product and had at least one post-baseline safety assessment.

For efficacy endpoints, changes from baseline to week 8 were compared between groups using a generalized linear model (GLM) with treatment group as a fixed effect and baseline value and age included as covariates. Least-squares (LS) mean differences with 95% confidence intervals (CIs) were presented for adjusted between-group comparisons. When data distributions were notably skewed or parametric assumptions were not appropriate, nonparametric Wilcoxon rank-sum tests were used for between-group comparisons. Within-group changes from baseline were assessed using paired *t*-tests or Wilcoxon signed-rank tests, as appropriate.

The primary endpoint was the change in NK cell activity from baseline to week 8 at effector-to-target (E:T) ratios of 50:1, 25:1, and 12.5:1. Secondary endpoints included changes in cytokines and IgG concentrations from baseline to week 8. Exploratory outcomes included changes in white blood cell count and differential percentages, changes in General Assessment of Recent Stress (GARS) scores, and upper respiratory tract infection (URTI) occurrence during the intervention period. Because NK cell activity was assessed at multiple prespecified E:T ratios and multiple secondary immune biomarkers were evaluated without formal multiplicity adjustment, *p*-values for individual outcomes should be interpreted cautiously and in the context of the overall consistency of findings.

When parametric assumptions were not met for between-group comparisons, *p*-values were derived from nonparametric Wilcoxon rank-sum tests. In such cases, adjusted least-squares (LS) mean differences and 95% confidence intervals from the generalized linear model are also presented to facilitate clinical interpretation of effect sizes, but the nonparametric *p*-value should be considered the primary inferential statistic for hypothesis testing.

Efficacy analyses were primarily based on observed cases within each analysis set. No formal multiple-imputation sensitivity analysis was performed. Therefore, differences between the mITT/FAS and PPS results should be interpreted with consideration of the potential influence of missing data, protocol deviations, and adherence-related attrition.

### 2.9. Participant Disposition

**Figure 1:**
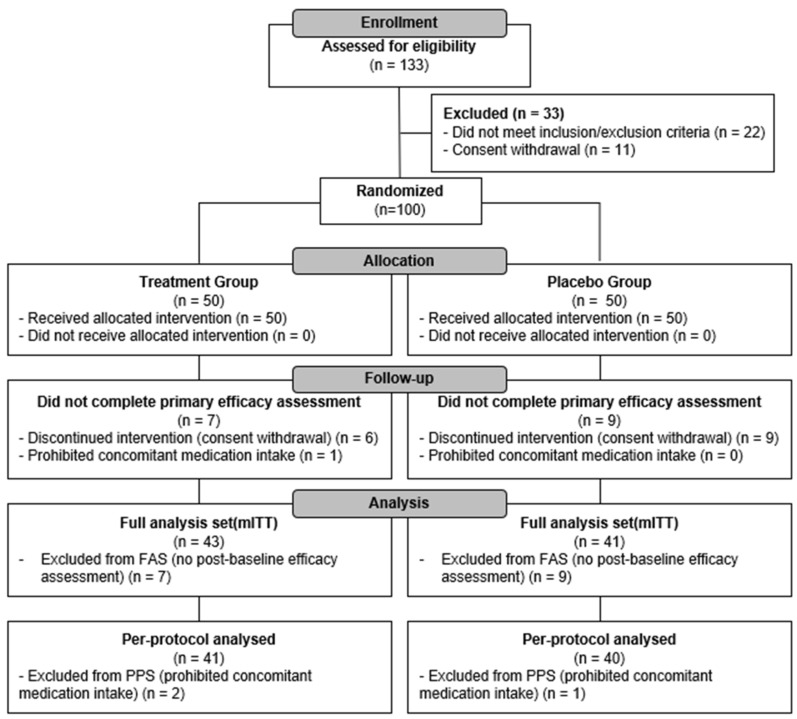
CONSORT flow diagram showing participant disposition, including enrollment, allocation, follow-up, and analysis populations (modified intention-to-treat/full analysis set and per-protocol set).

A total of 133 individuals were screened, of whom 33 were excluded during screening. One hundred participants were randomized in a 1:1 ratio to the fermented noni polysaccharide group or the placebo group (50 participants per group). Eighty-four participants completed the intervention and were included in the primary analysis set, the modified intention-to-treat/full analysis set (mITT/FAS; 43 in the fermented noni polysaccharide group and 41 in the placebo group). Three additional participants were excluded from the per-protocol set (PPS) because of major protocol deviations, resulting in 81 participants in the PPS sensitivity analysis population (41 in the treatment group and 40 in the placebo group) (Figure 1). The most common reasons for discontinuation were withdrawal of consent and use of prohibited concomitant products (Figure 1). Adherence, assessed by tablet counts, was high in both groups. Mean compliance exceeded 90% and did not differ meaningfully between groups.

## 3. Results

### 3.1. Baseline Characteristics

Baseline demographic and clinical characteristics are presented in Table 1. The fermented noni polysaccharide and placebo groups were comparable at baseline, with no significant differences in sex distribution, age, height, weight, or BMI. Mean age was approximately 50 years in both groups, and mean BMI was 25.47 ± 3.15 kg/m^2^ in the fermented noni polysaccharide group and 24.69 ± 3.65 kg/m^2^ in the placebo group. Additional baseline characteristics including lifestyle factors, URTI history, and baseline immune markers are presented in Table S4, with no significant between-group differences.

### 3.2. Primary Outcome: NK Cell Activity

At baseline, NK cell activity did not differ meaningfully between groups across the prespecified effector-to-target (E:T) ratios. In the primary mITT/FAS analysis, baseline- and age-adjusted between-group comparisons showed numerically greater but not statistically significant increases in NK cell activity in the fermented noni polysaccharide group compared with the placebo group at all three E:T ratios (Table 2). The adjusted least-squares (LS) mean differences (95% confidence intervals [CIs]) were 8.94 (−0.61 to 18.50; *p* = 0.0662) at E:T 50:1, 7.68 (−1.14 to 16.50; *p* = 0.0872) at E:T 25:1, and 3.29 (−2.95 to 9.54; *p* = 0.1447) at E:T 12.5:1. Specifically, NK cell cytotoxic activity in the treatment group increased from baseline to week 8 by mean changes of +10.32 ± 27.28, +11.46 ± 22.37, and +5.94 ± 13.73 percentage points at E:T ratios of 50:1, 25:1, and 12.5:1, respectively. In the placebo group, the corresponding mean changes were small and slightly negative (−0.59 ± 29.05, −2.94 ± 28.00, and −1.74 ± 19.76 percentage points). Thus, at all three E:T ratios, the within-group direction was upward in the treatment group and essentially flat or slightly downward in the placebo group, yielding consistent between-group LS mean differences in favour of FNP.

Although the treatment group showed numerically greater improvements than the placebo group, none of the adjusted between-group comparisons reached the conventional threshold for statistical significance in the primary mITT/FAS analysis. Because NK cell activity was assessed at three prespecified E:T ratios without formal multiplicity adjustment, these ratio-specific *p*-values should be interpreted cautiously and in the context of the overall pattern across ratios.

Prespecified PPS sensitivity analyses showed directionally consistent results, with larger between-group LS mean differences at the higher E:T ratios. In the PPS analysis, the adjusted LS mean differences (95% CIs) were 11.03 (1.46 to 20.61; *p* = 0.0245) at E:T 50:1, 9.94 (1.10 to 18.79; *p* = 0.0281) at E:T 25:1, and 4.26 (−2.14 to 10.66; *p* = 0.0864) at E:T 12.5:1 (Table S2). However, because PPS analyses are more susceptible to bias related to adherence, missing data, and protocol deviations, the primary interpretation of efficacy should be based on the mITT/FAS results.

### 3.3. Secondary Outcomes: Cytokines and IgG Levels

Changes in circulating cytokines and IgG from baseline to week 8 were evaluated in the primary mITT/FAS analysis. In baseline- and age-adjusted between-group analyses, several cytokines showed nominal differences between the fermented noni polysaccharide and placebo groups after 8 weeks of supplementation. The fermented noni polysaccharide group showed a greater increase in IL-6 than the placebo group, with an adjusted difference in change of 2.54 (95% CI 0.64 to 4.43; *p* = 0.0002). Nominal between-group differences were also observed for IFN-γ (0.37, 95% CI −0.02 to 0.77; *p* = 0.0496), IL-10 (0.06, 95% CI −0.01 to 0.13; *p* = 0.0312), and IL-1β (0.10, 95% CI −0.00 to 0.20; *p* = 0.0455), whereas IL-2 showed a borderline between-group difference (0.26, 95% CI −0.04 to 0.56; *p* = 0.0509). No meaningful between-group differences were observed for TNF-α, IL-12, or IgG (Table 3). Within-group, the treatment group exhibited mean increases from baseline to week 8 in IFN-γ (+0.87 pg/mL), IL-2 (+0.49 pg/mL), IL-6 (+1.94 pg/mL), IL-10 (+0.10 pg/mL), and IL-1β (+0.08 pg/mL), whereas in the placebo group these cytokines decreased slightly or remained essentially unchanged (IFN-γ −0.13 pg/mL, IL-2 −0.13 pg/mL, IL-6 −1.04 pg/mL, IL-10 −0.02 pg/mL, IL-1β −0.05 pg/mL). For TNF-α, IL-12, and IgG, the within-group changes were small and similar between groups, consistent with the absence of meaningful between-group differences.

Overall, although selected cytokines showed nominal between-group differences, the pattern was not uniform across all immune biomarkers, and no meaningful difference was observed for IgG. Given the exploratory nature of these biomarker analyses and the absence of formal multiplicity adjustment, these findings should be interpreted cautiously.

PPS analyses of cytokines and IgG are presented in the Supplementary Materials as sensitivity analyses (Table S3). These analyses may be useful for assessing directional consistency, but interpretation should remain centered on the primary mITT/FAS results.

### 3.4. Exploratory Outcomes

Total WBC count changed minimally from baseline to week 8 in both groups, with no significant between-group difference (Table S6). Neutrophil and lymphocyte percentages showed similar time-related shifts in both groups, and the magnitude of change did not differ significantly between groups (Table S6).

Changes in General Assessment of Recent Stress (GARS) scores differed between groups at week 4, whereas no significant between-group difference in change was observed at week 8 (Table S7). Given the exploratory nature of this outcome and the lack of persistence, the week 4 difference should be interpreted cautiously.

URTI occurrence during the 8-week intervention period was prespecified as an exploratory outcome and was assessed at week 4, week 8, and as a cumulative occurrence (Table 4). At week 4, URTI occurred in 5 of 43 participants (11.63%; 95% CI 2.05, 21.21) in the treatment group and 7 of 41 participants (17.07%; 95% CI 5.56, 28.59) in the placebo group (*p* = 0.4759, Chi-square test). At week 8, URTI occurred in 6 of 43 (13.95%; 95% CI 3.60, 24.31) in the treatment group and 4 of 41 (9.76%; 95% CI 0.67, 18.84) in the placebo group (*p* = 0.7387, Fisher’s exact test). Cumulative URTI occurrence over the 8-week intervention was 10/43 (23.26%) in the treatment group and 10/41 (24.39%) in the placebo group (*p* = 1.0000, Fisher’s exact test). The between-group differences in URTI occurrence did not reach statistical significance, which is consistent with the relatively brief 8-week observation window and the fact that the trial was not powered for clinical infection endpoints.

### 3.5. Safety

No clinically meaningful safety concerns were identified during the 8-week intervention. Laboratory assessments, including hematology, serum chemistry, and urinalysis, did not reveal any clinically meaningful between-group differences, and any within-group fluctuations remained within acceptable ranges. Hematology and serum chemistry results are presented in Table S8, and urinalysis findings are presented in Table S9. Overall, the investigational product was well tolerated, and the safety profile was comparable between groups. A total of 26 adverse events were reported in the treatment group and 24 in the placebo group during the 8-week intervention. The most frequently reported events in the treatment group were pharyngitis (8 events), dyspepsia (6 events), and headache (4 events); in the placebo group, the most frequently reported events were pharyngitis (6 events), headache (4 events), and dyspepsia (3 events). All adverse events were mild in severity, and the between-group difference in event frequency was not statistically significant. No serious adverse events occurred, no events were judged to be related to the investigational product or placebo, and no participant discontinued the trial because of an adverse event. A detailed listing of treatment-emergent adverse events is provided in Table S5.

## 4. Discussion

In this randomized, double-blind, placebo-controlled trial, fermented noni polysaccharides did not produce statistically significant between-group differences in NK cell activity in the primary mITT/FAS analysis, although the direction of effect was consistently favorable across the prespecified E:T ratios. Prespecified per-protocol analyses showed supportive findings at the higher E:T ratios, but these should be interpreted as sensitivity analyses rather than confirmatory evidence. Overall, the findings suggest a possible immunomodulatory signal, but they do not establish clinical efficacy. Collectively, the results should be interpreted cautiously in light of the non-significant primary mITT/FAS analysis, the supportive nature of the per-protocol findings, and the exploratory biomarker-focused design of the study.

NK cells are key effectors of early innate antiviral defence, mediating cytotoxic elimination of infected or stressed cells and contributing to immune orchestration via cytokines such as IFN-γ [16]. Inter-individual variability in NK activity is substantial, and lower NK cell activity has been associated with higher infection risk in observational settings, especially among older adults [20]. Therefore, the observed trend toward improved NK activity at higher E:T ratios, particularly evident in the PPS population with adequate adherence, may represent a directionally consistent biomarker signal of enhanced innate cytotoxic function. The absence of statistical significance at the lowest E:T ratio (12.5:1) may reflect reduced assay sensitivity under low effector pressure or greater biological variability, a pattern also observed in other nutritional interventions in which NK effects are more apparent at higher E:T conditions [19,21,22,23]. It should be noted that baseline NK cell activity was numerically lower in the treatment group than in the placebo group at E:T ratios of 25:1 and 12.5:1, although these differences were not statistically significant. Although baseline values were included as covariates in the generalized linear model to adjust for this imbalance, the possibility that regression to the mean contributed in part to the observed within-group improvement in the treatment group cannot be entirely excluded. This consideration further supports the need for replication in adequately powered trials.

The cytokine findings provide further context for the NK functional signal. IFN-γ is a central mediator of antiviral and anti-tumour immunity, and IL-2 supports NK activation, cytotoxic function, and T-cell expansion. Directionally coherent changes in IFN-γ and IL-2 levels together with the pattern of NK cell activity findings may be consistent with a possible cellular immune–leaning response; however, these cytokine findings remain secondary and exploratory and should be interpreted cautiously. Changes in IL-6 and IL-10 levels should be interpreted cautiously, given their context-dependent roles. IL-6 is a pleiotropic cytokine implicated in host defence and acute-phase responses that may reflect inflammatory activation depending on the setting [24]. In contrast, IL-10 is a key immunoregulatory cytokine that limits excessive inflammation and tissue injury [25]. The concurrent involvement of IL-10 may indicate immunomodulation that is not purely pro-inflammatory; however, cytokine kinetics were not captured beyond scheduled visits, and single-time-point measures cannot fully characterize temporal patterns. Previous nutritional intervention trials targeting immune function have similarly reported variable cytokine responses, underscoring the challenge of capturing clinically meaningful immune modulation with single-time-point assessments [26].

Among the cytokine findings, the increase in IL-6 warrants cautious interpretation. IL-6 is a pleiotropic, context-dependent cytokine involved in both immune regulation and inflammatory signalling, and persistently elevated levels are widely recognized as markers of low-grade inflammation and cardiometabolic risk. In the present study, IL-6 values showed substantial inter-individual variability, suggesting possible skewed distributions and heterogeneous responses. Because IL-6 was measured only at scheduled visits, the available data cannot determine whether the observed change reflects transient fluctuation, sustained elevation, or regression to the mean. Moreover, although the IL-6 change was not accompanied by concordant increases in TNF-α or IL-1β, clinically meaningful abnormalities in safety laboratory parameters, or adverse clinical findings, these observations do not establish a beneficial immunological effect. Therefore, given the exploratory nature of the cytokine analyses, the multiplicity context, and the mITT-based primary efficacy framework, the IL-6 finding should be regarded as hypothesis-generating and requires confirmation in future studies with denser longitudinal sampling and clinically relevant correlates. Notably, the substantial inter-individual variability in IL-6 values at baseline (SD exceeding the mean in the treatment group) raises the possibility that a small number of participants with high baseline values may have disproportionately influenced the group-level estimate. Future analyses should consider log-transformation or robust regression approaches, and sensitivity analyses excluding extreme values would help assess the robustness of this finding. By contrast, the concurrent changes in IFN-γ and IL-2 may be biologically consistent with the direction of the NK cell activity findings; however, these cytokine results should also be interpreted cautiously because they were secondary, exploratory outcomes and were not adjusted for multiplicity. Taken together, the cytokine profile may suggest a possible immunomodulatory signal rather than nonspecific inflammatory activation, but it does not establish clinical or mechanistic efficacy.

Mechanistically, the clinical findings are congruent with evidence that botanical polysaccharides can modulate innate immune pathways, often through pattern-recognition receptor signalling, leading to macrophage activation, nitric oxide signalling, and downstream cytokine cascades that can influence NK cell function [14,27]. In particular, polysaccharide recognition by Toll-like receptor 4 and Dectin-1 has been shown to activate NF-κB signalling and promote pro-inflammatory cytokine secretion in macrophages [28]. Preclinical studies of fermented *Morinda citrifolia* preparations report enhanced immune activity, including increased cytokine production and NK-related functional markers, supporting biological plausibility for the present observations [8,15]. Fermentation may alter polysaccharide composition and structural features (e.g., molecular weight distribution and branching), potentially affecting receptor engagement and bioactivity [5]; future work linking structural characterization with immune functional readouts would strengthen mechanistic understanding.

Routine hematology indices and WBC differentials are relatively insensitive markers of functional immune enhancement. In this study, WBC and differential proportions varied within groups over time, but between-group differences were not significant, suggesting that the primary signal is better captured by NK functional endpoints and cytokine dynamics rather than broad leukocyte redistribution. Psychological stress is another modifier of NK function; stress exposure has been associated with altered NK activity across stressor types and durations [29]. The inclusion of the GARS score provides contextual information relevant to immune responsiveness [30]. Nevertheless, residual confounding by unmeasured lifestyle factors (e.g., sleep quality, incidental infections, seasonal influences) cannot be fully excluded. Furthermore, cytokine measurements were obtained at predefined visits and do not capture temporal dynamics; therefore, longitudinal profiling would be required to fully characterize the immune response pattern.

Preclinical evidence in the Investigator’s Brochure provides additional context for the directional pattern observed in the present trial. In RAW 264.7 macrophages, fermented noni polysaccharide extract increased nitric oxide production and the secretion of IL-1β, IL-6, and TNF-α, together with upregulation of cyclooxygenase-2 and inducible nitric oxide synthase. In Balb/c mice, the same product increased NK cell activity and the production of IL-2, IL-12, IFN-γ, and IL-10, accompanied by increases in immune cell counts in immune-related organs. The directional consistency between these preclinical observations and the human findings—particularly the rise in NK cell activity together with concordant increases in IFN-γ, IL-2, IL-6, and IL-10—supports biological plausibility for an immunomodulatory effect, although the present clinical study did not directly assess receptor engagement, downstream signalling pathways, or gut microbiome modulation. The fermentation procedure (frozen *M. citrifolia* fruit fermented with *Levilactobacillus brevis* NST707, followed by 80-mesh filtration, concentration, ethanol precipitation, sterilisation, and freeze-drying) may modify polysaccharide composition, molecular-weight distribution, and bioavailability or recognition by innate immune cells, although the present study did not perform direct structural characterisation. Mechanistic interpretations should therefore be regarded as plausible inferences drawn from the preclinical literature on related polysaccharides rather than conclusions established by the present human data, and we have used cautious wording (e.g., “may suggest”, “may be associated with”) accordingly.

Several alternative explanations cannot be completely excluded for the observed biomarker patterns. These include regression to the mean (particularly given the slight numerical baseline imbalance in NK activity at the lower E:T ratios), chance findings due to multiple unadjusted comparisons, seasonal and incidental URTI exposure not measured during the trial, and unmeasured lifestyle confounders (e.g., sleep quality, dietary patterns, stress events) despite the randomized design. The fact that some endpoints (e.g., NK activity at the higher E:T ratios, IL-6, IL-10) showed greater between-group differences while others (TNF-α, IL-12, IgG, URTI incidence, GARS, WBC, leukocyte subsets) did not differ significantly highlights the heterogeneity of the response and underscores that the biomarker changes cannot be directly equated with clinical infection prevention. The observed cytokine variability—particularly for IL-6, IL-12, and IFN-γ, where standard deviations exceeded the mean—indicates that group-level estimates may have been influenced by a small number of participants with high baseline values; sensitivity analyses with log-transformation or robust regression in future studies would help assess the robustness of these signals. We also note that prespecified interaction analyses for variables such as age, sex, baseline NK activity, and URTI history were not formally performed because the trial was not powered to detect interaction effects; this is identified as an additional limitation, and any future signal in this regard should be regarded as hypothesis-generating. Overall, while the directional consistency of the immunological readouts is encouraging, the present results should be interpreted as exploratory biomarker findings and not as confirmation of clinical efficacy.

This study has several limitations. First, the primary endpoint was an immunologic biomarker (NK cell activity), and clinically adjudicated URTI outcomes (incidence, duration, and severity) were not primary endpoints, limiting direct inference regarding infection prevention. Second, the trial was registered at CRiS retrospectively on 12 December 2025, after trial completion, rather than prospectively before participant enrolment; although the IRB-approved protocol was finalised prior to recruitment and was not modified during the trial, the retrospective timing of registration limits transparency. Third, the primary mITT/FAS analysis did not reach statistical significance for NK cell activity, although point estimates were directionally consistent with the hypothesis and the PPS sensitivity analysis showed statistically significant effects at higher E:T ratios. The attenuation of effects in the mITT/FAS analysis compared with the PPS may reflect dilution due to non-adherent participants or protocol deviations, and the findings should be interpreted cautiously. Finally, NK cell activity was assessed at multiple prespecified E:T ratios and multiple cytokines were evaluated without a formal multiplicity-control strategy, increasing the risk of chance findings (type I error). Replication in larger, prospectively registered trials with prespecified estimands, sample-size justification, and hierarchical or other multiplicity-adjustment procedures is warranted. Evidence from trials and meta-analyses of probiotic/fermented dairy products suggests that fermented food interventions may reduce the incidence and/or burden of respiratory tract infections, supporting the inclusion of clinically adjudicated infection outcomes in future studies [31,32]. Additionally, biomarker-focused designs in nutritional immunology would benefit from standardised outcome frameworks and consensus-driven effect size benchmarks [26].

Despite these limitations, the present trial provides controlled human evidence suggesting that fermented noni polysaccharide supplementation may support NK cell activity and modulate selected immune-related cytokines while maintaining an acceptable safety profile.

## 5. Conclusions

In conclusion, 8-week supplementation with fermented noni polysaccharide extract showed numerically greater increases in NK cell activity at all three E:T ratios (50:1, 25:1, and 12.5:1) compared with placebo in the primary mITT/FAS analysis, although these between-group differences did not reach the conventional threshold for statistical significance. Prespecified PPS sensitivity analyses showed significant between-group differences at E:T ratios of 50:1 and 25:1. The treatment group also showed greater increases in selected cytokines (IFN-γ, IL-2, IL-6, IL-10) relative to placebo. No significant between-group differences were observed in URTI incidence, IgG, GARS, WBC, or leukocyte subset proportions. The intervention was well tolerated, with no serious adverse events or intervention-related adverse reactions. These exploratory biomarker findings suggest that fermented noni polysaccharide extract may have potential immunomodulatory effects, but further prospective, adequately powered studies with clinically adjudicated endpoints are needed to confirm its clinical relevance.

## Figures and Tables

**Table 1:** Baseline demographic and clinical characteristics.

	Treatment (N = 43)	Placebo (N = 41)	*p*-Value
Sex, *n* (%)			
Male	6 (13.95)	8 (19.51)	0.4944 (C)
Female	37 (86.05)	33 (80.49)	
Age (years)			
Mean ± SD	50.42 ± 7.96	50.66 ± 7.39	0.7811 (W)
Height (cm)			
Mean ± SD	160.26 ± 7.73	161.27 ± 7.26	0.5402 (T)
Weight (kg)			
Mean ± SD	65.52 ± 10.20	64.54 ± 12.43	0.4310 (W)
BMI			
Mean ± SD	25.47 ± 3.15	24.69 ± 3.65	0.1913 (W)

SD, standard deviation; BMI, body mass index; C, Chi-square test; W, Wilcoxon rank sum test; T, two-sample *t*-test.

**Table 2:** Change in NK cell activity from baseline to week 8 at E:T ratios 50:1, 25:1, and 12.5:1 (mITT/FAS).

Time Point	Group	50:1	25:1	12.5:1
Baseline	Placebo	48.11 ± 26.92	32.80 ± 25.36	16.82 ± 17.41
	Treatment	45.15 ± 26.80	23.63 ± 17.44	10.51 ± 9.97
Week 8	Placebo	47.52 ± 21.56	29.86 ± 18.16	15.08 ± 11.33
	Treatment	55.47 ± 25.20	35.09 ± 22.37	16.45 ± 17.01
Change	Placebo	−0.59 ± 29.05	−2.94 ± 28.00	−1.74 ± 19.76
	Treatment	10.32 ± 27.28	11.46 ± 22.37	5.94 ± 13.73
Between-group differences in change	Treatment—Placebo	8.94	7.68	3.29
	(95% CI)	(−0.61, 18.50)	(−1.14, 16.50)	(−2.95, 9.54)
	*p*-value	0.0662 (G)	0.0872 (G)	0.1447 (W)

Values are presented as mean ± SD unless otherwise indicated. N = 43 in the treatment group and N = 41 in the placebo group at all time points. Change represents the difference from baseline (Visit 2) to week 8 (Visit 4). Between-group differences are presented as least squares (LS) mean differences with 95% confidence intervals. *p*-values were calculated using a generalized linear model adjusted for baseline values and age (G) or the Wilcoxon rank-sum test (W), as indicated.

**Table 3:** Changes in cytokines and IgG from baseline to week 8 (mITT/FAS).

Cytokine	Time Point	Treatment	Placebo
IFN-γ (pg/mL)	Baseline	3.24 ± 8.45	2.09 ± 1.04
	Week 8	4.11 ± 13.16	1.96 ± 0.84
	Change	0.87 ± 4.75	−0.13 ± 0.61
	*p*-value *	0.1108 (W)	0.1324 (W)
	Difference in Change (95% CI)	0.37 (−0.02, 0.77)	
	*p*-value ^ŧ^	0.0496 (W)	
TNF-α (pg/mL)	Baseline	2.01 ± 0.73	1.99 ± 0.64
	Week 8	2.10 ± 1.14	1.96 ± 0.48
	Change	0.09 ± 0.57	−0.03 ± 0.38
	*p*-value *	0.5849 (W)	0.6174 (W)
	Difference in Change (95% CI)	0.12 (−0.09, 0.33)	
	*p*-value ^ŧ^	0.4518 (W)	
IL-2 (pg/mL)	Baseline	3.08 ± 5.96	2.31 ± 0.74
	Week 8	3.57 ± 8.73	2.18 ± 0.70
	Change	0.49 ± 2.81	−0.13 ± 0.57
	*p*-value *	0.4208 (W)	0.0797 (W)
	Difference in Change (95% CI)	0.26 (−0.04, 0.56)	
	*p*-value ^ŧ^	0.0509 (W)	
IL-6 (pg/mL)	Baseline	5.27 ± 16.17	4.10 ± 4.91
	Week 8	7.21 ± 23.83	3.06 ± 2.55
	Change	1.94 ± 8.18	−1.04 ± 3.13
	*p*-value *	0.0058 (W)	0.0086 (W)
	Difference in Change (95% CI)	2.54 (0.64, 4.43)	
	*p*-value ^ŧ^	0.0002 (W)	
IL-10 (pg/mL)	Baseline	0.65 ± 0.93	0.52 ± 0.17
	Week 8	0.75 ± 1.34	0.50 ± 0.15
	Change	0.10 ± 0.43	−0.02 ± 0.12
	*p*-value *	0.0270 (W)	0.3379 (T)
	Difference in Change (95% CI)	0.06 (−0.01, 0.13)	
	*p*-value ^ŧ^	0.0312 (W)	
IL-12 (pg/mL)	Baseline	3.45 ± 7.04	2.47 ± 1.34
	Week 8	4.44 ± 13.75	2.33 ± 0.65
	Change	0.99 ± 6.75	−0.14 ± 1.22
	*p*-value *	0.5976 (W)	0.9161 (W)
	Difference in Change (95% CI)	0.25 (−0.56, 1.06)	
	*p*-value ^ŧ^	0.6947 (W)	
IL-1β (pg/mL)	Baseline	1.02 ± 1.65	0.76 ± 0.29
	Week 8	1.09 ± 1.87	0.72 ± 0.21
	Change	0.08 ± 0.32	−0.05 ± 0.20
	*p*-value *	0.2117 (W)	0.1422 (T)
	Difference in Change (95% CI)	0.10 (−0.00, 0.20)	
	*p*-value ^ŧ^	0.0455 (W)	
IgG (µg/mL)	Baseline	2247.75 ± 683.37	2132.51 ± 526.20
	Week 8	2218.40 ± 656.80	2105.61 ± 504.88
	Change	−29.35 ± 376.50	−26.90 ± 271.73
	*p*-value *	0.9559 (W)	0.5298 (T)
	Difference in Change (95% CI)	18.25 (−117.02, 153.52)	
	*p*-value ^ŧ^	0.7137 (W)	

Values are presented as mean ± SD unless otherwise indicated. N = 43 in the treatment group and N = 41 in the placebo group at all time points. Change represents the difference from baseline (Visit 2) to Week 8 (Visit 4). Between-group differences are presented as LS mean differences with 95% confidence intervals. *: *p*-values were calculated using a paired *t*-test (T) or the Wilcoxon signed-rank test (W), as appropriate. ^ŧ^: *p*-values were calculated using a generalized linear model adjusted for baseline values and age (G) or the Wilcoxon rank-sum test (W), as appropriate. IFN, interferon; IL, interleukin; TNF, tumour necrosis factor; Ig, immunoglobulin; LS, least-squares mean; BL, baseline; W8, week 8. Note: Adjusted LS mean differences and 95% CIs from the GLM (adjusted for baseline values and age) are presented for effect-size interpretation, whereas the Wilcoxon rank-sum test *p*-values are used as the primary inferential statistics because the cytokine and IgG distributions did not satisfy parametric assumptions, consistent with the prespecified analysis plan.

**Table 4:** URTI occurrence at week 4, week 8, and cumulative during the 8-week intervention (mITT/FAS).

Time Point	Treatment (N = 43)	Placebo (N = 41)	*p*-Value
Week 4 occurrence, *n* (%)	5 (11.63)	7 (17.07)	0.4759 (C)
(95% CI)	(2.05, 21.21)	(5.56, 28.59)	
Week 8 occurrence, *n* (%)	6 (13.95)	4 (9.76)	0.7387 (F)
(95% CI)	(3.60, 24.31)	(0.67, 18.84)	
Cumulative occurrence, *n* (%)	10 (23.26)	10 (24.39)	1.0000 (F)
(95% CI)	(10.63, 35.88)	(11.25, 37.54)	

95% CI, two-sided 95% confidence interval. Between-group *p*-values were calculated using the Chi-square test (C) or Fisher’s exact test (F), as appropriate.

## Data Availability

The data presented in this study are available on reasonable request from the corresponding author. The data are not publicly available due to ethical restrictions and the protection of participant privacy.

## Supplementary Materials

The following supporting information can be downloaded at https://www.mdpi.com/article/10.3390/nu18111691/s1. Table S1: Eligibility Criteria (Inclusion and Exclusion); Table S2: Change in NK cell activity from baseline to week 8 at E:T ratios 50:1, 25:1, and 12.5:1 (PPS); Table S3: Changes in cytokines and IgG from baseline to week 8 (PPS); Table S4: Baseline characteristics including immune markers, lifestyle factors, and URTI history (mITT/FAS); Table S5: Treatment-emergent adverse events (TEAEs) summary (Safety Set); Table S6: WBC Count and Differential Changes from Baseline to Week 8 (mITT/FAS); Table S7: General Assessment of Recent Stress (GARS) Score over 8 Weeks (mITT/FAS); Table S8: Hematology and Clinical Chemistry Safety Parameters at Baseline and Week 8 (Safety Set); Table S9: Urinalysis Findings at Baseline and Week 8 (Safety Set). Figure S1: Study design and visit schedule for the 8-week randomized, double-blind, placebo-controlled trial of fermented noni polysaccharides versus placebo. Participants attended four visits: screening (Visit 1), baseline/randomization (Visit 2, Day 0), mid-intervention assessment (Visit 3, Day 28 ± 5), and end-of-intervention assessment (Visit 4, Day 56 ± 5); Figure S2: Appearance of the investigational product (treatment) and placebo tablets (left) and representative HPLC chromatogram with retention time and galacturonic acid (GalA) content of FNP (right). Galacturonic acid (GalA) was quantified by HPLC after acid hydrolysis (2 M trifluoroacetic acid, 100 °C, 8 h) and PMP derivatization, using a Shimadzu LC system (LC-20AC pumps, SPD-M20A detector) with a Waters X-Bridge C18 column (4.6 × 250 mm, 5 µm). Mobile phases: (A) 10% acetonitrile in 100 mM sodium acetate buffer (pH 5.0) and (B) 20% acetonitrile in the same buffer; gradient 50:50 (*v*/*v*) for 2 min then 100% B to 50 min, 1.0 mL/min, 40 °C, UV 245 nm, 10 µL injection. The GalA content of the FNP extract was 76.10 mg/g (specification ± 20%); The representative chromatogram value (78.02 ± 0.28 mg/g) falls within this specification range.

## Abbreviations

The following abbreviations are used in this manuscript:

AE(s)	Adverse event(s)
ANCOVA	Analysis of covariance
BMI	Body mass index
BL	Baseline
CI	Confidence interval
CONSORT	Consolidated Standards of Reporting Trials
E:T	Effector-to-target (ratio)
GARS	General Assessment of Recent Stress
GLM	Generalized linear model
Hb	Haemoglobin
HDL	High-density lipoprotein (cholesterol)
IFN-γ	Interferon-gamma
IgG	Immunoglobulin G
IL	Interleukin (e.g., IL-2, IL-6, IL-10, IL-12, IL-1β)
LS mean	Least-squares mean
NK	Natural killer
PPS	Per-protocol set
SD	Standard deviation
TNF-α	Tumour necrosis factor-alpha
ULN	Upper limit of normal
URTI	Upper respiratory tract infection
WBC	White blood cell
