# Supplementary material for: Fermented Noni Polysaccharides and Immune-Related Biomarkers in Adults with Recurrent URTIs: A Randomized, Double-Blind, Placebo-Controlled Trial
Source: Nutrients. 2026 May 26;18(11):1691. doi: 10.3390/nu18111691 (PMC13258254; doi:10.3390/nu18111691)
Supplement: Supplementary file 1 [file nutrients-18-01691-s001.zip › nutrients-4267886-supplementary.pdf]

Table S1. Eligibility Criteria (Inclusion and Exclusion)

**Inclusion Criteria**	
**1**	Men and women aged 40 to <75 years at screening.
**2**	Peripheral white blood cell (WBC) count at screening: ≥4.0 × 10^3 and <8.0 × 10^3 cells/μL.
**3**	History of ≥2 episodes of upper respiratory tract infection (URTI) within 1 year prior to screening (e.g., common cold, tonsillitis, pharyngitis, laryngitis, sinusitis, otitis media, rhinitis, etc.).
**4**	Provided written informed consent and voluntarily signed the consent form.

**Exclusion Criteria**	
**1**	Within 1 month before screening (or at screening), having a disease/condition that may affect immune function (e.g., infectious diseases, autoimmune diseases, leukaemia, immune thrombocytopenia, malignancy, liver disease including active hepatitis, metabolic syndrome, systemic inflammatory diseases, marked malnutrition, etc.).
**2**	Within 1 month prior to screening, use or current use of health functional foods/supplements that may affect immunity (e.g., vitamins, probiotics, ginseng/red ginseng, or other immune-supporting supplements).
**3**	Within 3 months prior to screening, use or current use of immunosuppressants, antibiotics, non-steroidal anti-inflammatory drugs (NSAIDs), steroid medications, or antihistamines.
**4**	Received vaccination within 3 months prior to screening.
**5**	Confirmed COVID-19 infection within 3 months prior to screening.
**6**	Uncontrolled hypertension (blood pressure ≥160/100 mmHg after 10 mins of rest at screening).
**7**	Uncontrolled diabetes (fasting glucose ≥126 mg/dL or HbA1c ≥6.5%), or initiation/dose change of antidiabetic medication within 3 months prior to screening.
**8**	Serum creatinine >2.0 mg/dL at screening.
**9**	AST or ALT ≥3× the upper limit of normal (ULN) of the testing institution at screening.
**10**	Currently under treatment for severe cardiovascular, immune, respiratory, hepatobiliary, renal/urinary, neurologic, musculoskeletal, psychiatric, infectious diseases, or malignancy.
**11**	Within 3 months prior to screening: myocardial infarction, coronary artery bypass grafting, percutaneous coronary intervention (e.g., angioplasty or stent), active unstable angina, heart failure (NYHA class III–IV), severe valvular disease/defect (e.g., aortic or mitral stenosis), stroke, or untreated atrial fibrillation.
**12**	Gastrointestinal diseases that may affect absorption of the investigational product (e.g., Crohn's disease, chronic dyspepsia, irritable bowel syndrome), or a history of gastrointestinal surgery within 6 months prior to screening.
**13**	Thyroid disease (thyroid dysfunction/symptoms or abnormal thyroid function tests).

**14**	Based on the alcohol-habit questionnaire at screening: average alcohol intake during the past month of ≥210 g/week (men) or ≥140 g/week (women).
**15**	Heavy smoker (≥20 cigarettes/day).
**16**	Pregnant, lactating, or planning pregnancy during the study period.
**17**	Known hypersensitivity or allergy to any ingredient of the investigational product.
**18**	Participation in a similar clinical trial within 3 months prior to screening.
**19**	Any other reason judged by the investigator to make the participant ineligible.

Abbreviations: AST, aspartate transferase; ALT, alanine transferase; WBC, white blood cell; URTI, upper respiratory tract infection; ULN, upper limit of normal; NSAIDs, non-steroidal anti-inflammatory drugs; NYHA, New York Heart Association.

Table S2. Change in NK cell activity from baseline to week 8 at E:T ratios 50:1, 25:1, and 12.5:1 (PPS)

Time point	Group	50:1	25:1	12.5:1
Baseline	Placebo	48.11±27.26	33.56±25.21	17.20±17.47
	Treatment	44.28±27.16	23.88±17.71	10.72±10.14
Week 8	Placebo	46.55±20.91	29.17±17.82	14.80±11.32
	Treatment	56.13±25.56	36.13±22.39	17.01±17.20
Change (BL→W8)	Placebo	−1.56±28.74	−4.40±26.73	−2.40±19.54
	Treatment	11.85±26.92	12.25±22.56	6.29±13.97
Between-group differences in change	Treatment – Placebo	11.03	9.94	4.26
	(95% CI)	(1.46, 20.61)	(1.10, 18.79)	(−2.14, 10.66)
	P-value	0.0245 (G)	0.0281 (G)	0.0864 (W)

Values are presented as mean ± SD unless otherwise indicated. N = 41 in the treatment group and N = 40 in the placebo group at all time points. Change represents the difference from baseline (Visit 2) to Week 8 (Visit 4). Between-group differences are presented as LS mean differences with 95% confidence intervals. *P*-values were calculated using a generalized linear model adjusted for baseline values and age (G) or the Wilcoxon rank-sum test (W), as indicated.

Table S3. Changes in cytokines and IgG from baseline to week 8 (PPS)

Cytokine	Time Point	Treatment	Placebo
IFN-γ	Baseline	3.30±8.66	2.11±1.05
(pg/mL)	Week 8	4.23±13.47	1.93±0.83

	Change (BL→W8)	0.93±4.85	-0.18±0.53
	P-value*	0.0617 (W)	0.0489 (W)
	Differences of Change (95% CI)	0.46(0.07, 0.85)	
	P-valueᵗ	0.0179 (W)	
TNF-α (pg/mL)	Baseline	2.00±0.74	2.00±0.64
	Week 8	2.09±1.17	1.94±0.47
	Change (BL→W8)	0.09±0.58	-0.06±0.35
	P-value*	0.6876 (W)	0.4555 (W)
	Differences of Change (95% CI)	0.15(-0.06, 0.35)	
	P-valueᵗ	0.3760 (W)	
IL-2 (pg/mL)	Baseline	3.12±6.11	2.31±0.75
	Week 8	3.63±8.94	2.13±0.62
	Change (BL→W8)	0.51±2.88	-0.18±0.46
	P-value*	0.4211 (W)	0.0325 (W)
	Differences of Change (95% CI)	0.32(0.03, 0.61)	
	P-valueᵗ	0.0312 (W)	
IL-6 (pg/mL)	Baseline	5.43±16.55	4.17±4.96
	Week 8	7.49±24.38	3.07±2.58
	Change (BL→W8)	2.06±8.37	-1.10±3.15
	P-value*	0.0017 (W)	0.0031 (W)
	Differences of Change (95% CI)	2.68(0.73, 4.64)	
	P-valueᵗ	<0.0001 (W)	
IL-10 (pg/mL)	Baseline	0.66±0.95	0.53±0.16
	Week 8	0.76±1.37	0.50±0.15
	Change (BL→W8)	0.10±0.44	-0.03±0.10
	P-value*	0.0569 (W)	0.1180 (T)
	Differences of Change (95% CI)	0.07(0.00, 0.14)	
	P-valueᵗ	0.0299 (W)	
IL-12	Baseline	3.52±7.21	2.49±1.35

(pg/mL)	Week 8	4.56±14.07	2.32±0.66
	Change (BL→W8)	1.05±6.91	-0.17±1.22
	P-value[*]	0.7070 (W)	0.6813 (W)
	Differences of Change (95% CI)	0.30(-0.54, 1.14)	
	P-value[ŧ]	0.8864 (W)	
IL-1β	Baseline	1.03±1.69	0.77±0.29
(pg/mL)	Week 8	1.10±1.92	0.71±0.20
	Change (BL→W8)	0.08±0.33	-0.06±0.18
	P-value[*]	0.2117 (W)	0.1422 (T)
	Differences of Change (95% CI)	0.11(0.01, 0.21)	
	P-value[ŧ]	0.0516 (W)	
IgG	Baseline	2219.93±685.37	2109.50±511.58
(μg/mL)	Week 8	2216.68±671.61	2091.23±502.74
	Change (BL→W8)	-3.26±351.15	-18.27±269.44
	P-value[*]	0.6727 (W)	0.6704 (T)
	Differences of Change (95% CI)	28.54(-103.68, 160.76)	
	P-value[ŧ]	0.6401 (W)	

Values are presented as mean ± SD unless otherwise indicated. N = 41 in the treatment group and N = 40 in the placebo group at all time points. Change represents the difference from baseline (Visit 2) to Week 8 (Visit 4). Between-group differences are presented as LS mean differences with 95% confidence intervals. [*]: *P*-values were calculated using a Paired t-test (T) or the Wilcoxon signed rank test (W), as appropriate. [ŧ]: *P*-values were calculated using a generalized linear model adjusted for baseline values and age (G) or the Wilcoxon rank-sum test (W), as appropriate. Note: Adjusted LS mean differences and 95% CIs from the GLM are provided for effect-size interpretation, whereas Wilcoxon rank-sum test p-values are used as the primary inferential statistics for cytokines and IgG because the distributions did not satisfy parametric assumptions, consistent with the manuscript Table 3 footnote and the prespecified analysis plan. IFN, interferon; IL, interleukin; TNF, tumour necrosis factor; Ig, immunoglobulin; LS, least-squares mean; BL, baseline; W8, week 8.

Table S4. Baseline characteristics including immune markers, lifestyle factors, and URTI history (mITT/FAS)

**Variable**		**Placebo (N = 41)**	**Treatment (N = 43)**	***P*-value**
**Lifestyle factors**				
**Smoking status, n (%) Never**	•	37 (90.24)	39 (90.70)	0.4366 (F)
**Smoking status, n (%) Former**	•	2 (4.88)	0 (0.00)	

**Smoking status, n (%)**<br>**• Current**	2 (4.88)	4 (9.30)	
**Smoking exposure (pack-years)**	—	—	—
**Alcohol intake, n (%)**<br>**• None**	18 (43.90)	19 (44.19)	1.0000 (F)
**Alcohol intake, n (%)**<br>**• Former (quit)**	1 (2.44)	2 (4.65)	
**Alcohol intake, n (%)**<br>**• Current**	22 (53.66)	22 (51.16)	
**Exercise habit ≥3 times/week, n (%)**	29 (70.73)	24 (55.81)	0.1567 (C)
**URTI history (prior 12 months)**			
**Participants with ≥1 URTI episode, n (%)**	41 (100.00)	43 (100.00)	—
**Number of URTI episodes (count)**	≥2 episodes (eligibility)	≥2 episodes (eligibility)	—
**Days with URTI symptoms (days) (if available)**	—	—	—
**Antibiotic use for URTI, n (%) (if available)**	—	—	—
**Questionnaire**			
**GARS total score (0–84)**	20.80±11.66	20.02±8.44	0.9037 (W)
**Baseline NK cell activity**			
**NK cell activity (%) at E:T = 50:1**	48.11±26.92	45.15±26.80	0.5311 (W)
**NK cell activity (%) at E:T = 25:1**	32.80±25.36	23.63±17.44	0.1682 (W)
**NK cell activity (%) at E:T = 12.5:1**	16.82±17.41	10.51±9.97	0.1682 (W)
**Baseline cytokines & immunoglobulin**			
**IFN-γ (pg/mL)**	2.09±1.04	3.24±8.45	0.8157 (W)
**IL-2 (pg/mL)**	2.31±0.74	3.08±5.96	0.6351 (W)
**IL-12 (pg/mL)**	2.47±1.34	3.45±7.04	0.9144 (W)
**IL-6 (pg/mL)**	4.10±4.91	5.27±16.17	0.5942 (W)
**IL-1β (pg/mL)**	0.76±0.29	1.02±1.65	0.6770 (W)

**IL-10 (pg/mL)**	0.52±0.17	0.65±0.93	0.9144 (W)
**TNF-α (pg/mL)**	1.99±0.64	2.01±0.73	0.8957 (W)
**IgG (μg/mL)**	2132.51±526.20	2247.75±683.37	0.2429 (W)

Data are presented as mean ± SD for approximately normally distributed continuous variables; median (IQR) may be used if distributions are skewed. Categorical variables are presented as n (%). P values may be calculated using two sample t-test(T) (or Wilcoxon rank sum test(W)) for continuous variables and χ^2 test(C) (or Fisher's exact test(F)) for categorical variables. Baseline was defined as the pre-intervention assessment. Detailed cumulative smoking exposure (pack-years), URTI symptom duration in days, and antibiotic use for URTI were not systematically collected during the trial and are therefore not available; only the screening-based eligibility criterion of ≥2 documented URTI episodes within the prior 12 months was recorded for all participants. This limitation is also noted in the Discussion.

Table S5. Treatment-emergent adverse events (TEAEs) summary (Safety Set)

Summary category	Placebo (N = 50)	Treatment (N = 50)	*P*-value
**Participants with ≥1 TEAE, n (%)**	17 (34.00)	19 (38.00)	0.6769
**Total number of TEAEs, n**	24	26	—
**Participants with treatment-related TEAE, n (%)**	0 (0.00)	0 (0.00)	—
**Participants with ≥1 serious AE (SAE), n (%)**	0 (0.00)	0 (0.00)	—
**Participants discontinued due to AE, n (%)**	0 (0.00)	0 (0.00)	—
**Grade 1 (mild) TEAEs, n**	24	26	—
**Grade 2 (moderate) TEAEs, n**	0	0	—
**Grade ≥3 (severe) TEAEs, n**	0	0	—

TEAE, treatment-emergent adverse event. Safety Set includes all participants who received at least one dose of study product. Counts are number of participants experiencing ≥1 event in the category (not number of events), unless otherwise specified. Severity may be graded per study SOP (e.g., mild/moderate/severe). Relatedness reflects investigator assessment.

Table S6. WBC Count and Differential Changes from Baseline to Week 8 (mITT/FAS)

Outcome	Time Point	Treatment	Placebo
WBC ($\times10^3/\mu L$)	Baseline	5.92±1.06	5.59±1.02
	Week 8	5.62±1.33	5.42±0.99
	Change (BL→W8)	-0.30±1.08	-0.17±0.78
	P-value*	0.0770 (T)	0.1704 (T)
	Differences of Change (95% CI)	-0.03(-0.43, 0.37)	

	P-value[ŧ]	0.8770 (G)	
Neutrophils (%)	Baseline	52.59±8.57	52.45±9.02
	Week 8	55.29±6.98	55.27±8.16
	Change (BL→W8)	2.70±7.95	2.82±5.75
	P-value*	0.0314 (T)	0.0032 (T)
	Differences of Change (95% CI)	-0.10(-2.57, 2.38)	
	P-value[ŧ]	0.9367 (G)	
Lymphocyte (%)	Baseline	36.79±8.14	36.82±8.54
	Week 8	34.47±6.64	34.91±7.79
	Change (BL→W8)	-2.31±7.25	-1.91±4.91
	P-value*	0.0427 (T)	0.0168 (T)
	Differences of Change (95% CI)	-0.37(-2.58, 1.85)	
	P-value[ŧ]	0.7418 (G)	

Values are presented as mean ± SD unless otherwise indicated. n = 43 in the treatment group and n = 41 in the placebo group at all time points. Change represents the difference from baseline (Visit 2) to Week 8 (Visit 4). Between-group differences are presented as LS mean differences with 95% confidence intervals. *: *P*-values were calculated using a Paired t-test(T) or the Wilcoxon signed rank test(W), as appropriate. ŧ: *P*-values were calculated using a generalized linear model adjusted for baseline values and age(G) or the Wilcoxon rank-sum test(W), as appropriate. WBC, white blood cell; mITT, modified intention-to-treat ; LS, least-squares mean; BL, baseline; W8, week 8.

Table S7. General Assessment of Recent Stress (GARS) Score over 8 Weeks (mITT/FAS)

Time Point	Treatment	Placebo
Baseline	20.02±8.44	20.80±11.66
Week 4	20.72±9.96	17.85±8.62
Change (BL→W4)	0.70±7.36	-2.95±7.46
P-value*	0.5376 (T)	0.0153 (T)
Differences of Change (95% CI)	3.35(0.49, 6.21)	
P-value[ŧ]	0.0223 (G)	
Week 8	19.28±8.88	18.49±10.79
Change (BL→W8)	-0.74±6.12	-2.32±8.84
P-value*	0.4299 (T)	0.0721 (W)
Differences of Change (95% CI)	1.30(-1.72, 4.33)	
P-value[ŧ]	0.3196 (W)	

Values are presented as mean ± SD unless otherwise indicated. N = 43 in the treatment group and N = 41 in the placebo group at all time points. Change represents the difference from baseline (Visit 2) to Week 4 (Visit 3) and Week 8 (Visit 4). Between-group differences are presented as LS mean differences with 95% confidence intervals. [*]: *P*-values were calculated using a Paired t-test (T) or the Wilcoxon signed rank test (W), as appropriate. [ŧ]: *P*-values were calculated using a generalized linear model adjusted for baseline values and age (G) or the Wilcoxon rank-sum test (W), as appropriate. GARS, General Assessment of Recent Stress; mITT, modified intention-to-treat ; LS mean, least-squares mean ; BL, baseline; W4, week 4; W8, week 8.

Table S8. Hematology and Clinical Chemistry Safety Parameters at Baseline and Week 8 (Safety Set)

Domain	Parameter (unit)	Group	Baseline (Visit 1) Mean±SD	Baseline (Visit 1) Median (Min, Max)	Week 8 (Visit 4) Mean±SD	Week 8 (Visit 4) Median (Min, Max)	*P*-value (within-group)
Haematology	WBC (×10³/μL)	Treatment	5.96±1.05	5.97 (4.06, 7.85)	5.62±1.33	5.51 (3.21, 10.17)	0.0770 (T)
		Placebo	5.69±1.02	5.70 (4.14, 7.89)	5.42±0.99	5.45 (3.87, 8.29)	0.1704 (T)
	RBC (×10⁶/μL)	Treatment	4.59±0.37	4.56 (3.76, 5.71)	4.59±0.33	4.58 (3.87, 5.42)	0.5851 (T)
		Placebo	4.65±0.46	4.62 (3.69, 5.76)	4.59±0.44	4.49 (3.84, 5.80)	0.8273 (T)
	Hb (g/dL)	Treatment	13.41±1.64	13.60 (8.60, 17.00)	13.42±1.33	13.50 (9.20, 16.50)	0.8068 (W)
		Placebo	13.77±1.93	13.85 (9.30, 18.50)	13.39±1.90	13.50 (9.50, 17.60)	0.3255 (T)
	Hct (%)	Treatment	39.87±4.11	40.45 (28.90, 50.30)	39.74±3.34	39.80 (31.00, 48.50)	0.9385 (W)
		Placebo	40.94±5.17	41.10 (29.90, 54.70)	39.91±4.94	39.50 (30.30, 51.70)	0.3040 (T)
	Platelets (×10³/μL)	Treatment	283.56±52.89	273.00 (197.00, 451.00)	282.74±63.67	271.00 (205.00, 492.00)	0.8644 (T)
		Placebo	262.24±63.46	253.50 (116.00, 405.00)	266.73±62.52	267.00 (119.00, 433.00)	0.1449 (T)
	Neutrophil (%)	Treatment	52.39±8.84	52.35 (32.30, 69.80)	55.29±6.98	56.80 (40.70, 71.40)	0.0314 (T)

		Placebo	52.15±8.51	51.70 (31.90, 69.20)	55.27±8.16	57.50 (37.50, 70.00)	0.0032 (T)
	Lymphocyte (%)	Treatment	37.12±8.39	38.00 (20.70, 55.60)	34.47±6.64	34.30 (19.20, 47.70)	0.0427 (T)
		Placebo	37.10±8.30	36.75 (20.00, 57.90)	34.91±7.79	33.70 (22.80, 53.20)	0.0168 (T)
Clinical chemistry (Liver function)	AST (GOT) (U/L)	Treatment	24.50±5.38	25.00 (14.00, 36.00)	24.14±8.14	23.00 (14.00, 65.00)	0.5680 (W)
		Placebo	24.20±6.01	23.00 (15.00, 41.00)	23.07±6.35	22.00 (14.00, 45.00)	0.2916 (T)
	ALT (GPT) (U/L)	Treatment	21.88±11.63	19.00 (7.00, 68.00)	20.26±10.38	18.00 (7.00, 58.00)	0.7615 (W)
		Placebo	20.18±8.98	18.50 (5.00, 46.00)	18.93±10.25	16.00 (7.00, 65.00)	0.4189 (W)
	γ-GTP (U/L)	Treatment	19.80±12.25	16.00 (8.00, 64.00)	19.58±16.55	16.00 (8.00, 115.00)	0.9464 (W)
		Placebo	29.28±34.79	17.00 (7.00, 171.00)	21.20±16.45	15.00 (7.00, 79.00)	0.9926 (W)
	Total bilirubin (mg/dL)	Treatment	0.75±0.35	0.68 (0.30, 2.11)	0.76±0.40	0.70 (0.30, 2.52)	0.6011 (W)
		Placebo	0.79±0.45	0.72 (0.26, 2.62)	0.80±0.39	0.70 (0.33, 2.49)	0.8074 (T)
	Total protein (g/dL)	Treatment	7.35±0.37	7.40 (6.60, 8.10)	7.33±0.36	7.30 (6.40, 8.00)	0.8647 (T)
		Placebo	7.36±0.34	7.40 (6.70, 8.30)	7.38±0.40	7.30 (6.60, 8.30)	0.2667 (T)
	Albumin (g/dL)	Treatment	4.31±0.24	4.30 (4.00, 4.90)	4.31±0.26	4.30 (3.90, 4.90)	0.7857 (W)
		Placebo	4.30±0.24	4.30 (3.70, 4.90)	4.29±0.24	4.20 (3.80, 4.70)	0.8970 (T)
Clinical chemistr	Total cholesterol (mg/dL)	Treatment	188.46±28.60	189.00 (127.00, 272.00)	195.77±28.44	196.00 (128.00, 273.00)	0.2032 (T)

y (Lipids)		Placebo	201.34±41.43	202.50 (117.00, 312.00)	207.15±41.89	210.00 (119.00, 316.00)	0.0499 (W)
	Triglyceride (mg/dL)	Treatment	132.88±76.63	108.00 (57.00, 426.00)	142.49±80.36	115.00 (41.00, 407.00)	0.1582 (W)
		Placebo	134.28±54.04	124.00 (56.00, 293.00)	129.37±50.13	121.00 (54.00, 292.00)	0.5720 (T)
	HDL cholesterol (mg/dL)	Treatment	59.34±13.21	57.00 (29.00, 95.00)	62.47±13.71	62.00 (37.00, 97.00)	0.2321 (T)
		Placebo	63.68±15.67	63.50 (39.00, 111.00)	64.15±12.65	61.00 (46.00, 99.00)	0.3324 (T)
	LDL cholesterol (mg/dL)	Treatment	114.38±30.63	114.00 (26.00, 205.00)	120.09±26.01	120.00 (65.00, 189.00)	0.2710 (T)
		Placebo	124.68±34.90	126.50 (61.00, 213.00)	130.73±38.16	127.00 (59.00, 213.00)	0.0474 (W)
Clinical chemistry (Renal)	BUN (mg/dL)	Treatment	13.44±2.96	13.65 (6.80, 20.00)	13.80±4.06	14.00 (6.90, 22.50)	0.7232 (T)
		Placebo	13.17±4.17	12.80 (5.30, 28.80)	13.30±4.00	12.50 (6.70, 25.90)	0.8149 (T)
	Creatinine (Cr) (mg/dL)	Treatment	0.75±0.14	0.71 (0.52, 1.26)	0.73±0.14	0.69 (0.52, 1.31)	0.1574 (W)
		Placebo	0.80±0.14	0.78 (0.58, 1.17)	0.77±0.15	0.73 (0.53, 1.31)	0.0816 (T)
Clinical chemistry (Metabolic)	Glucose (mg/dL)	Treatment	86.82±8.37	86.50 (69.00, 106.00)	87.81±10.75	85.00 (65.00, 125.00)	0.5231 (W)
		Placebo	89.54±7.13	88.00 (78.00, 109.00)	89.29±9.34	89.00 (70.00, 119.00)	0.8309 (W)
	HbA1c (%)	Treatment	5.56±0.35	5.60 (5.00, 6.40)	5.56±0.34	5.60 (5.00, 6.50)	0.3079 (W)
		Placebo	5.55±0.29	5.50 (4.90, 6.30)	5.55±0.24	5.50 (5.10, 6.20)	0.5889 (W)

Values are presented as mean ± SD and median (min, max). The Safety Set included all randomized participants who received at least one dose of the study product (N = 50 per group). For safety laboratory assessments, baseline corresponds to Visit 1 (screening), whereas efficacy baseline assessments were defined at Visit 2 (randomization/baseline). Baseline laboratory values were available for all 50 participants per group; Week 8 measurements were available for 43 participants in the treatment group and 41 in the placebo group due to study discontinuations. Within-group comparisons were performed on participants with paired data at both time points. Baseline corresponds to Visit 1, and Week 8 corresponds to Visit 4. *P*-values are for within-group comparisons (Baseline vs Week 8) using the paired t-test (T) or Wilcoxon signed-rank test (W), as indicated. WBC, white blood cell; RBC, red blood cell; HbA1c, glycated haemoglobin; BUN, blood urea nitrogen; Cr, creatinine; HDL, high density cholesterol; LDL, low density cholesterol; AST, aspartate transferase; ALT, alanine transferase; SD, standard deviation; SE, standard error; Hb, haemoglobin; Hct, haematocrit.

Table S9. Urinalysis Findings at Baseline and Week 8 (Safety Set)

**Parameter**	**Group**	**Baseline status**	**Week 8 Normal, n (%)**	**Week 8 Abnormal, n (%)**	***P*-value (within-group)†**
Specific gravity	Treatment (N = 43)	Normal	39 (90.70)	1 (2.33)	0.3173
		Abnormal	3 (6.98)	0 (0.00)	
	Placebo (N = 41)	Normal	39 (95.12)	1 (2.44)	0.3173
		Abnormal	0 (0.00)	1 (2.44)	
pH	Treatment (n=43)	Normal	43 (100.0)	0 (0.00)	-
		Abnormal	0 (0.00)	0 (0.00)	
	Placebo (n=41)	Normal	41 (100.0)	0 (0.00)	-
		Abnormal	0 (0.00)	0 (0.00)	
Protein	Treatment (n=43)	Normal	35 (81.40)	6 (13.95)	0.0588
		Abnormal	1 (2.33)	1 (2.33)	
	Placebo (n=41)	Normal	34 (82.93)	4 (9.76)	0.7055
		Abnormal	3 (7.32)	0 (0.00)	
Glucose	Treatment (n=43)	Normal	43 (100.0)	0 (0.00)	-
		Abnormal	0 (0.00)	0 (0.00)	
	Placebo (n=41)	Normal	41 (100.0)	0 (0.00)	-
		Abnormal	0 (0.00)	0 (0.00)	
Blood (RBC)	Treatment (n=43)	Normal	26 (60.47)	3 (6.98)	0.1317
		Abnormal	8 (18.60)	6 (13.95)	
	Placebo (n=41)	Normal	26 (63.41)	6 (14.63)	0.7630
		Abnormal	5 (12.20)	4 (9.76)	

Values are presented as n (%). Analysis set: SAS. Week 8 measurements were available for 43 participants in the treatment group and 41 participants in the placebo group. † *P*-values are for within-group comparisons (Baseline vs Week 8) using McNemar's test. "-" indicates that the *p*-value was not estimable (e.g., no discordant pairs or no change in status).

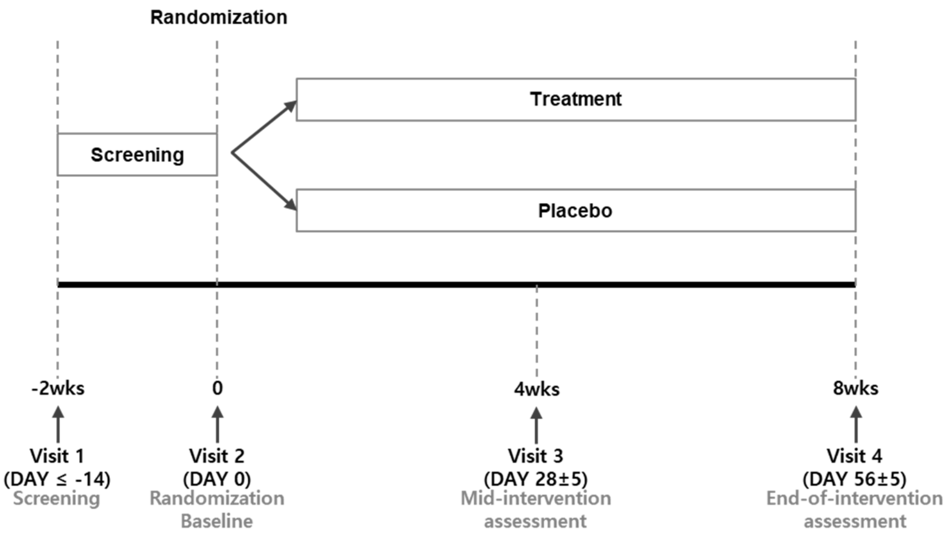

Figure S1. Study design and visit schedule for the 8-week randomized, double-blind, placebo-controlled trial of fermented noni polysaccharides versus placebo. Participants attended four visits: screening (Visit 1), baseline/randomization (Visit 2, Day 0), mid-intervention assessment (Visit 3, Day 28 ± 5), and end-of-intervention assessment (Visit 4, Day 56 ± 5).

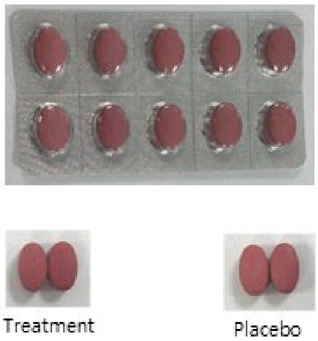

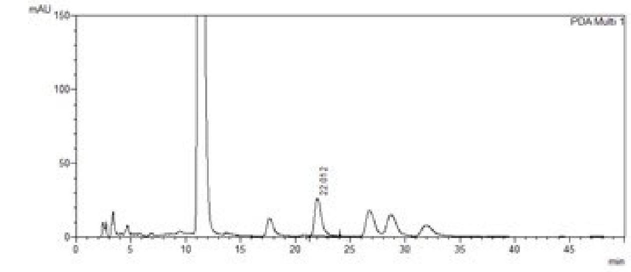

Retention time and content of FNP components	
	Galacturonic acid (GalA)
N	3
Retention time (min)	Standard: 21.933<br>FNP: 22.012
Contents (Mean±SD, mg/g)	78.02±0.28

Figure S2. Appearance of the investigational product (treatment) and placebo tablets (left) and representative HPLC chromatogram with retention time and galacturonic acid (GalA) content of FNP (right).

Galacturonic acid (GalA) was quantified by HPLC after acid hydrolysis (2 M trifluoroacetic acid, 100 °C, 8 h) and PMP derivatization, using a Shimadzu LC system (LC-20AC pumps, SPD-M20A detector) with a Waters X-Bridge C18 column (4.6 × 250 mm, 5 µm). Mobile phases: (A) 10% acetonitrile in 100 mM sodium acetate buffer (pH 5.0) and (B) 20% acetonitrile in the same buffer;

gradient 50:50 (v/v) for 2 min then 100% B to 50 min, 1.0 mL/min, 40 °C, UV 245 nm, 10 μL injection. The GalA content of the FNP extract was 76.10 mg/g (specification ± 20%); The representative chromatogram value (78.02 ± 0.28 mg/g) falls within this specification range.
